# Elevated serum IL-18 and IL-37 levels and their association with seizure burden in paediatric epilepsy

**DOI:** 10.3389/fimmu.2026.1883876

**Published:** 2026-08-17

**Authors:** Marta Zawadzka, Jolanta Gleń, Przemysław Waszak, Marta Pietruszka, Anna Lemska, Magdalena Trzeciak, Maria Mazurkiewicz-Bełdzińska

**Affiliations:** 1 Department of Developmental Neurology, Medical University of Gdańsk, University Clinical Center, Gdańsk, Poland; 2 Department of Dermatology, Venereology and Allergology, Medical University of Gdańsk, University Clinical Center, Gdańsk, Poland; 3 Division of Hygiene & Epidemiology, Medical University of Gdańsk, Gdańsk, Poland

**Keywords:** biomarker, Interleukin-18, Interleukin-37, neuroinflammation, paediatric epilepsy

## Abstract

**Background:**

Neuroinflammation is increasingly recognized as a key contributor to epileptogenesis and epilepsy progression. Interleukin-18 (IL-18), a pro-inflammatory cytokine of the interleukin-1 family, has been implicated in seizure susceptibility, whereas interleukin-37 (IL-37) acts as a natural anti-inflammatory regulator of IL-18 driven response. However, the role of the IL-18/IL-37 axis in paediatric epilepsy remains unclear.

**Objectives:**

The aim of this study was to assess serum IL-18 and IL-37 levels and to examine their associations with seizure burden in paediatric epilepsy.

**Methods:**

A total of 117 children with epilepsy and 53 age- and sex-matched healthy controls were included. Serum concentrations of IL-18 and IL-37 were measured using enzyme-linked immunosorbent assay. IL-18 and IL-37 gene polymorphisms were analyzed by allele-specific polymerase chain reaction.

**Results:**

Both IL-18 and IL-37 levels were significantly elevated in patients with epilepsy compared with controls with the highest concentrations observed in children with high seizure frequency (P vs controls: IL-18 p = 0.0022; IL-37 p ≤ 0.0050). A strong positive correlation was observed between IL-18 and IL-37 concentrations (R = 0.75, p < 0.0001). No significant associations were found between cytokine levels and epilepsy duration, time since the last seizure or other clinical parameters. Genetic polymorphisms did not differ significantly between patients and controls.

**Conclusions:**

Our findings suggest that IL-18 and IL-37 are associated with seizure burden and may represent promising candidate biomarkers in paediatric patients with epilepsy. However, these findings should be considered preliminary and warrant confirmation in larger independent longitudinal studies. To our knowledge, this is the first clinical study to assess serum IL-37 concentrations in patients with epilepsy.

## Highlights

- First clinical evidence of IL-37 involvement in paediatric epilepsy
- IL-18 and IL-37 levels increase with seizure frequency
- Strong correlation suggests coordinated inflammatory regulation
- IL-18/IL-37 axis may reflect inflammatory changes associated with seizure burden.

## 1 Introduction

The prevalence of active epilepsy is estimated at approximately 6.38/1000 individuals, affecting more than 51 million people worldwide. Although many patients achieve seizure freedom following initiation of therapy, nearly one-third develop drug-resistant epilepsy despite multiple treatment attempts (1, 2). In 2010, the International League Against Epilepsy (ILAE) defined drug-resistant epilepsy as the failure of 2 appropriately chosen and tolerated anti-seizure medications (ASMs) to achieve sustained seizure freedom (3). This heterogeneous group of patients often experiences frequent seizures that significantly impair daily functioning and neurodevelopment. As current treatment strategies remain largely symptomatic and frequently insufficient, identifying novel therapeutic targets presents as a major clinical priority (4).

Recent evidence has established neuroinflammation as a pathophysiological mechanism in epilepsy. Neuroinflammatory pathways are now recognized not only as contributors to seizure generation but also as potential therapeutic targets in epilepsy, particularly in drug-resistant patients (5). Neuroinflammation involves complex interactions between innate and adaptive immune responses, including activation of microglia, astrocytes, and endothelial cells, as well as recruitment of peripheral immune cells and release of inflammatory factors (5). While initially protective, persistent or dysregulated neuroinflammation may promote neuronal hyperexcitability and creates a self-sustaining cycle of seizures and inflammatory activation (5, 6).

The inflammatory cytokines of the interleukin-1 family play a prominent role in epileptogenesis (7). Interleukin-1β (IL-1β) has been widely studied in this context, acting through the IL-1 receptor type 1 (IL-1R1) to activate pro-inflammatory signaling cascades, whereas the IL-1 receptor antagonist (IL-1RA) counteracts these effects and exhibits anti-convulsant properties in experimental models (7). These findings highlight the IL-1 signaling axis as a key regulator of seizure susceptibility. The IL-1 cytokine family comprises 11 ligands and 10 receptors, including interleukin-18 (IL-18) and interleukin-37 (IL-37) (8). However, the roles of IL-18 and IL-37 in epilepsy, particularly in paediatric populations, remain insufficiently understood (9).

IL-18 was originally identified as an interferon-γ (IFN-γ)-inducing factor due to its ability to stimulate IFN-γ production in T helper 1 (Th1) cells (10). Signaling through IL-18 receptors, which contain a conserved Toll/interleukin-1 receptor (TIR) domain, activates downstream pathways such as nuclear factor-κB (NF-κB) and mitogen-activated protein kinases (MAPKs). IL-18 is produced by multiple cell types and plays a central role in innate immune responses by amplifying danger signals. Its activation requires caspase-1-mediated processing within the NOD-like receptor family, pyrin domain containing 3 (NLRP3) inflammasome, although non-canonical activation via caspase-8 and other proteases has also been described (11). Experimental studies suggest that IL-18 may contribute to neurodegeneration and exert proconvulsive effect, while under certain conditions it may also exert neuroprotective actions (12). Elevated serum IL-18 levels have been reported in adult epilepsy. However, data in paediatric epilepsy is limited (13).

IL-18 activity is regulated by IL-37, an anti-inflammatory cytokine that binds to the IL-18 receptor α chain and cooperates with IL-18 binding protein to suppress IFN-γ production by natural killer cells. Elevated IL-37 expression has been documented in several autoimmune diseases (e.g. rheumatoid arthritis, systemic lupus erythematosus), whereas its role in epilepsy, particularly in children and adolescents, has not yet been explored (14, 15).

Considering the lack of sufficient clinical data on IL-37 in epilepsy, the present study aimed to evaluate serum concentrations of IL-18 and IL-37 and to investigate their associations with seizure burden in paediatric patients with epilepsy. Additionally, we assessed selected IL-18 and IL-37 gene polymorphisms to explore potential genetic contributions to interindividual variability in cytokine expression. We hypothesized that IL-18 and IL-37 may serve as biomarkers associated with seizure burden in children.

## 2 Materials and methods

The study was designed as a single-center, prospective, observational study, conducted between September 2023 and September 2024.

### 2.1 Study population

A total of 136 patients, diagnosed with epilepsy according to the ILAE operational clinical definition of epilepsy (16) (study group) were recruited from the Developmental Neurology Clinic, along with 54 healthy individuals (control group, group Z).

Three subgroups were identified within the study cohort, based on seizure frequency during the previous 3 months of stable anti-seizure treatment: patients with high seizure frequency (≥8 seizures/month, subgroup P), patients with infrequent seizures (<8 seizures/month, subgroup S), and patients with seizure freedom for ≥12 months (subgroup B). The cut off of ≥8 seizures/month was selected for study stratification to distinguish patients with high seizure burden from those with lower seizure frequency.

Patients included in the study were aged between 1 and 17 years. Informed consent was obtained from all participating patients aged 16 years and older as well as all patients’ legal guardians.

Patients with coexisting autoimmune diseases, active infection or taking any anti-inflammatory medications/drugs or those, who did not provide informed consent, were excluded from the study.

### 2.2 Sample collection

Blood samples were collected from all participants in the morning hours and processed according to standardized protocols. Samples were collected under fasting conditions and prior to the administration of morning medications. Blood samples were allowed to clot at room temperature and centrifuged at 1500 x g for 10 minutes. Serum was separated, aliquoted and stored at -80 C until analysis.

### 2.3 Cytokine measurement

The concentrations of IL-18 and IL-37 in serum were measured with commercially available enzyme-linked immunosorbent assays kit - IL-18 ELISA SEA064Hu, IL-37 ELISA SEE842Hu; Cloud-Clone Corp. (USA), Kit ordered by Immuniq, Warsaw, Poland. This product has been tested by Quality Control and complied with internal specifications. Samples were assayed in duplicate, with the mean concentration value calculated. The provided standards and positive controls were used for testing. Standard curve concentrations for IL-18 ranged from 0 (blank) to 1000 pg/mL. For IL-37, from 0 (blank) to 500 pg/mL. Assay sensitivity was defined as the lowest concentration different from zero. All samples were assayed in duplicate, with the mean concentration value calculated. All procedures were performed according to the manufacturer’s protocol. Measurements were performed using a microplate reader (ELx800, manufactured by BioTek) at a wavelength of 450 nm.

### 2.4 Genotyping

DNA samples were isolated from peripheral blood with the Blood Mini DNA extraction kit, according to the manufacturer’s instructions (A&A Biotechnology, Gdañsk, Poland). The analysis of polymorphic variants of IL-37 gene (A/G rs3811047) and -137G/C IL-18 gene promoter was performed by the amplification refractory mutation system – polymerase chain reaction method (ARMS-PCR) using designed specific sequence primers (PCR-SSP). The growth hormone gene GH1 served as an internal amplification control.

### 2.5 Ethical approval

The Independent Bioethics Committee for Scientific Research at Medical University of Gdańsk approved this study (NKBBN/553/2022-2023) and the study was conducted in accordance with the Declaration of Helsinki.

### 2.6 Statistical analysis

Clinical data were obtained from the hospital’s information system and organized in a spreadsheet. Data were collected in Microsoft Excel (Microsoft Corporation, Redmond, WA, USA, file format: Office Open XML, Excel 2007) and analyzed using the Statistica software (version 10.0, TIBCO Software Inc., Palo Alto, USA). Normality was assessed with the Kolmogorov–Smirnov test. Depending on distribution, appropriate parametric or nonparametric tests were applied. Group comparisons used the t-test or Mann–Whitney U test for two groups, and ANOVA or Kruskal–Wallis test for multiple groups. Categorical data were analyzed using Pearson’s chi-square test with Yates’ correction when appropriate. Statistical significance was set at p < 0.05.

Data distributions for correlations between ILs were first assessed for normality as well. As the normality assumption was not met, non-parametric methods were applied for subsequent analyses. Relationships between variables were evaluated using Spearman’s rank correlation coefficient, as well as additional non-parametric measures (Kendall’s Tau and Goodman–Kruskal Gamma) to account for potential monotonic associations without assuming linearity or normal distribution of the data.

The effect size for the Mann–Whitney U test was calculated as the r coefficient (r = Z/√N), with values around 0.10 interpreted as a small effect, around 0.30 as a moderate effect, and ≥ 0.50 as a large effect. For the Kruskal–Wallis test, the effect size was expressed as the epsilon squared (ϵ²) coefficient, with values around 0.01 considered a small effect, around 0.08 a moderate effect, and ≥ 0.26 a large effect.

## 3 Results

### 3.1 Study population characteristics

**Figure 1:**
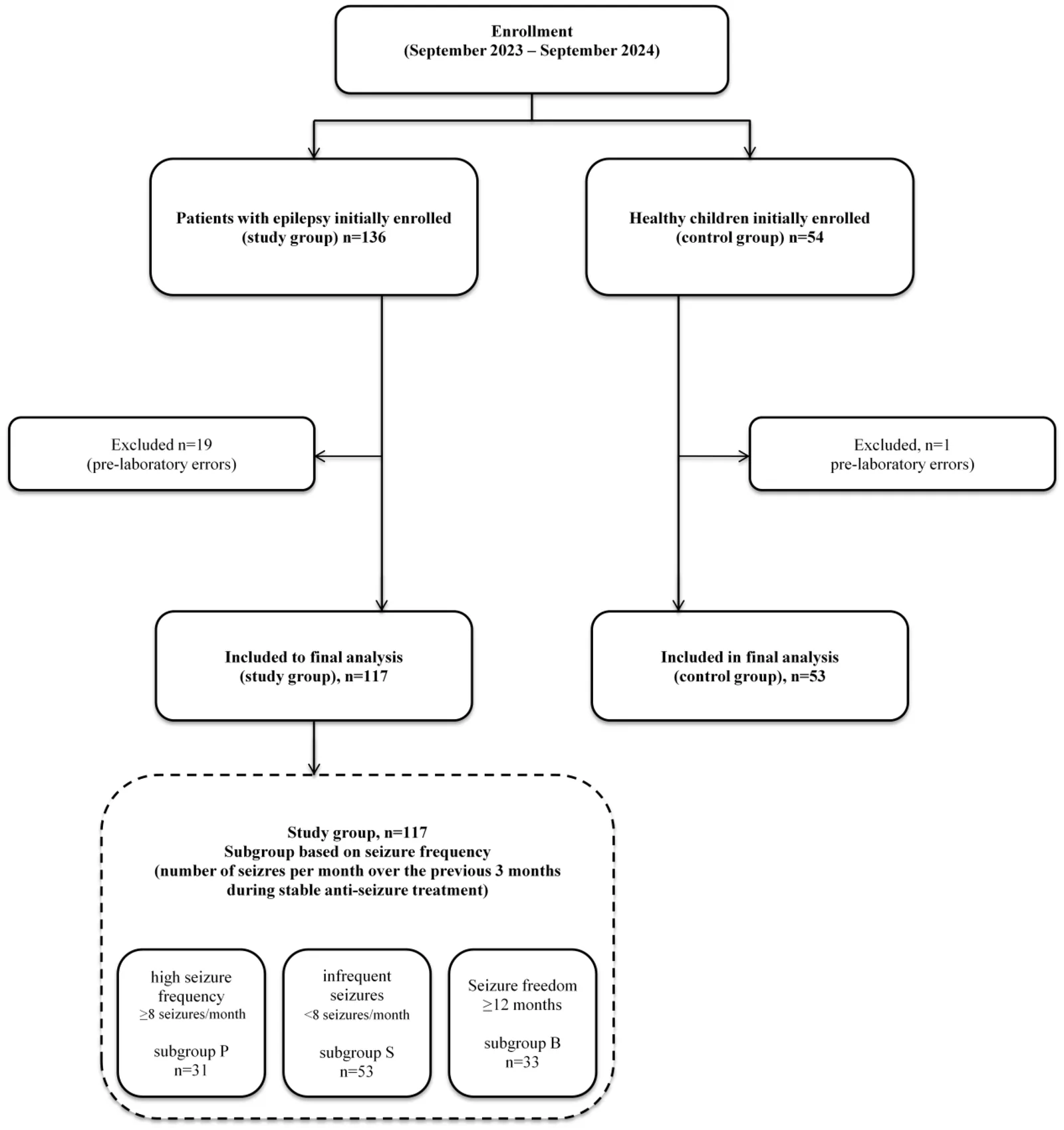
Flow diagram of participant enrollment, exclusion and final study population.

A total of 170 children (91 males, 53.5%; 79 females, 46.5%**),** 1–17 years of age were included in the final statistical analysis (**Figure 1**). This cohort comprised of 53 healthy controls (group Z) and 117 patients with epilepsy (31 in subgroup P, 53 in subgroup S, 33 in subgroup B) (**Table 1**). Twenty participants were excluded due to pre-laboratory errors (e.g. hemolysis, inadequate sample volume, mislabeling).

**Table 1:** Clinical and demographic characteristics of the study cohort.

Variable	P (n = 31)	S (n = 53)	B (n = 33)	Z (n = 53)	P value
Age (years)	8.79 ± 5.15 9 (4–13)	8.12 ± 4.70 7 (5–13)	9.73 ± 4.01 10 (7–12)	9.40 ± 4.70 9 (5–14)	0.355
Male sex, n (%)	20 (64.5)	27 (50.9)	16 (48.5)	28 (52.8)	0.573
Age at epilepsy onset (years)	2.81 ± 3.09 2 (1–4)	4.35 ± 3.93 3 (1.5–5)	5.73 ± 3.42 5 (3–8)	–	< 0.001
Duration of epilepsy (years)	5.97 ± 4.62 4 (2–11)	3.49 ± 3.74 2 (0.5–6)	4.88 ± 3.70 3 (2–6)	–	0.014
Adverse pregnancy/perinatal history, n (%)	11 (35.5)	11 (20.8)	4 (12.1)	0	0.160
Delayed developmental milestones, n (%)	20 (64.5)	14 (26.4)	4 (12.1)	0	< 0.001
EEG findings, n (%)					0.007
— Generalized abnormalities	13 (41.9)	11 (20.8)	3 (9.1)	0	
— Focal abnormalities	9 (29.0)	18 (34.0)	3 (9.1)	0	
— Normal EEG	8 (25.8)	23 (43.4)	25 (75.8)	0	
— Slow activity	1 (3.2)	1 (1.9)	2 (6.1)	0	
Seizure type, n (%)					0.358
— Generalized motor	16 (51.6)	20 (37.7)	11 (33.3)	0	
— Generalized non-motor	1 (3.2)	5 (9.4)	1 (3.0)	0	
— Focal motor	2 (6.5)	12 (22.6)	9 (27.3)	0	
— Focal non-motor	10 (32.3)	16 (30.2)	12 (36.4)	0	
— Polymorphic	2 (6.5)	0	0	0	
Mean seizure duration (min)	2.40 ± 2.80 2 (0.5–3)	2.35 ± 2.92 2 (1–3)	1.53 ± 0.92 2 (0.5–2)	–	0.541
Interval since last seizure (days)	7.9 ± 17.5 1 (1–7)	72.1 ± 81.7 30 (14–120)	912 ± 549 730 (365–1460)	–	< 0.001
Seizures per month	168.6 ± 336.6 28 (8–150)	1.83 ± 2.16 1 (0.33–2)	0 ± 0 0 (0–0)	–	< 0.001
Number of anti-seizure medications	2.45 ± 0.72 3 (2–3)	1.45 ± 0.77 1 (1–2)	1.36 ± 0.70 1 (1–2)	–	< 0.001
Clobazam treatment, n (%)	12 (38.7)	12 (22.6)	0	0	0.002
Valproate treatment, n (%)	16 (51.6)	23 (43.4)	13 (39.4)	0	0.799
Carbamazepine treatment, n (%)	7 (22.6)	11 (20.8)	7 (21.2)	0	0.998
Intellectual disability, n (%)	23 (74.2)	18 (34.0)	6 (18.2)	2 (3.8)	< 0.001
Etiology, n (%)					0.047
- Unknown	11 (37.9)	34 (66.7)	28 (84.8)	0	
- Genetic	8 (27.6)	6 (11.8)	1 (3.0)	0	
- Structural	12 (34.5)	11 (21.5)	4 (11.2)	0	

Data are presented as mean ± standard deviation and median (interquartile range: Q1–Q3), or n (%).

P – epilepsy with ≥8 seizures/month; S – epilepsy with ≤7 seizures/month; B – seizure-free for >12 months; Z – healthy controls.

Mean age (± SD) was 8.79 ± 5.15 years in subgroup P, 8.12 ± 4.70 years in subgroup S, 9.73 ± 4.01 years in subgroup B, and 9.40 ± 4.70 years in group Z. There were no significant differences in age, sex or seizure types (generalized motor, generalized non-motor, focal motor, focal non-motor, polymorphic) between the subgroups.

### 3.2 Clinical features according to seizure burden

Seizure-related variables showed strong group effects. The interval since the last seizure increased from group P (7.9 ± 17.5 days) to S (72.1 ± 81.7 days) and was longest in group B (912 ± 549 days; p < 0.0001). Monthly seizure frequency differed markedly (P: 168.6 ± 336.6; S: 1.83 ± 2.16; B: 0; p < 0.0001). Intellectual disability was most prevalent in group P (74.2%), followed by S (34.0%) and B (18.2%), whereas it was rare in controls (3.8%; p < 0.0001).

The number of ASMs was highest in subgroup P (2.45 ± 0.72**),** compared with subgroup S (1.45 ± 0.77) and subgroup B (1.36 ± 0.70) p < 0.0001. Overall, 45.5% of patients were on monotherapy and 54.5% on polytherapy. No significant differences were observed between the subgroups regarding the use of valproic acid (VPA) or carbamazepine (CBZ). A significant difference was found for clobazam (CLB) use (p = 0.002), which was observed in subgroups P and S but not in subgroup B.

In the full cohort, no correlation was found between IL-18 and IL-37 levels and clinical features such as age, age at epilepsy onset, epilepsy duration, time since the last seizure or the number of ASMs (all p *>* 0.05).

### 3.3 Association between ASMs and cytokine levels

Serum IL-37 concentrations differed significantly depending on ASM use, with lower levels observed in patients treated with VPA compared with those not receiving VPA (p = 0.028), and higher levels in patients treated with CBZ compared with untreated patients (p = 0.006), whereas no significant differences were found for clobazam. In contrast, serum IL-18 concentrations did not differ significantly with respect to treatment with VPA, CBZ, or clobazam.

### 3.4 Serum IL-18 and IL-37 levels

**Figure 2:**
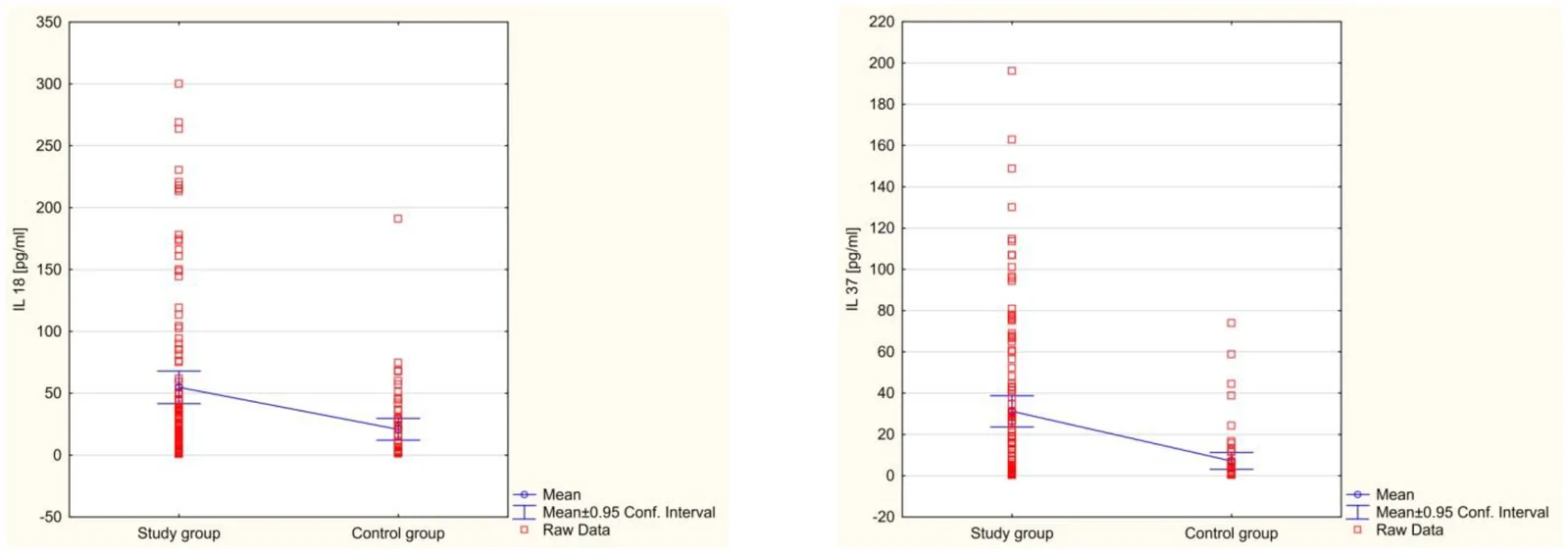
Levels of IL-18 and IL-37 in patients with epilepsy and in the control group. Horizontal lines indicate median values. Statistical significance was assessed using the Mann–Whitney U test (p = 0.00063 for IL-18; p < 0.0001 for IL-37).

The results demonstrated that both IL-18 and IL-37 levels were significantly elevated in epilepsy patients (**Figure 2**). Mean IL-18 concentrations were 54.84 ± 70.19 pg/mL in the epilepsy group and 20.92 ± 31.73 pg/mL in the control group (p = 0.00063, r = 0.266). Mean IL-37 concentrations were 31.18 ± 40.51 pg/mL in the epilepsy group and 7.17 ± 14.86 pg/mL in the control group (p < 0.0001, r = 0.306).

### 3.5 Cytokine levels and seizure burden

**Figure 3:**
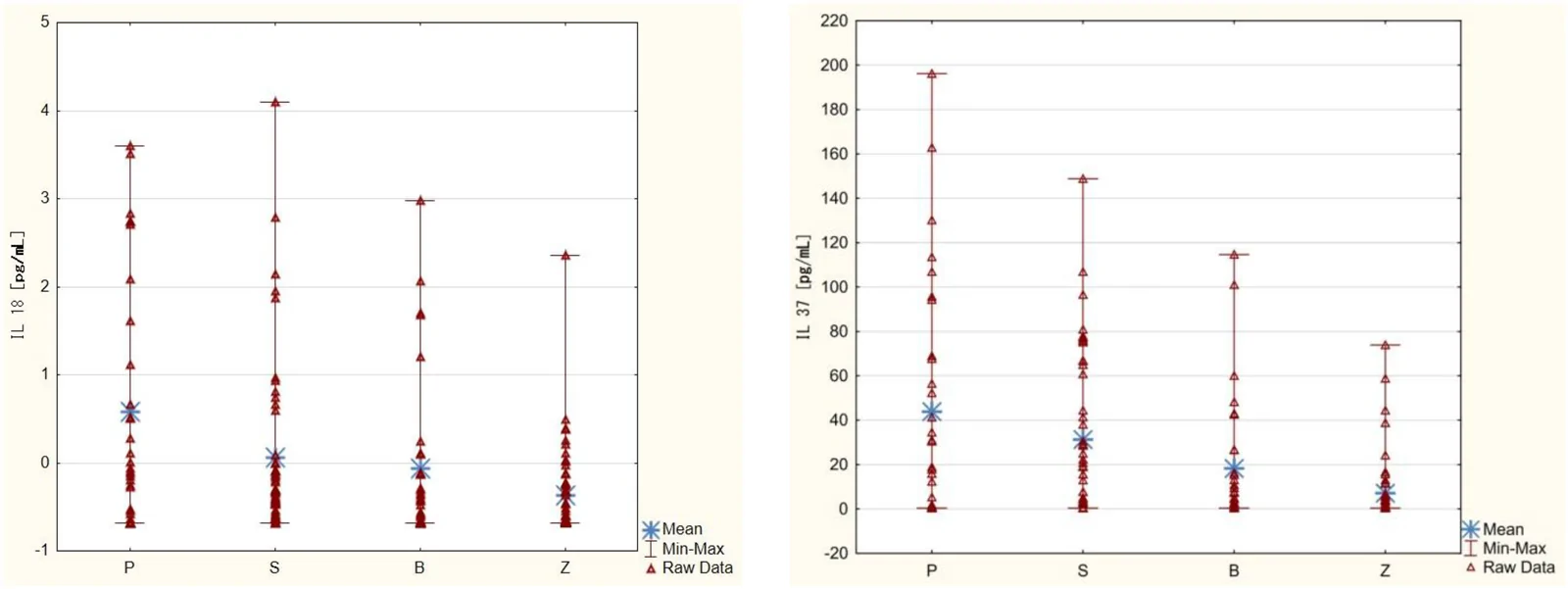
Concentrations of IL-18 and IL-37 according to seizure burden (P - ≥8 seizures/month, S - <8 seizures/month, B - patients with seizure freedom for ≥12 months, Z - healthy individuals). Overall group differences were assessed using the Kruskal–Wallis test (IL-18: *p* = 0.0010; IL-37: *p* < 0.001).

The concentrations of IL-18 and IL-37 were associated with the seizure burden. The highest levels were observed in patients with ≥8 seizures/month (subgroup P), while the lowest were found in patients who achieved complete seizure freedom with ASMs (subgroup B) (**Figure 3**).

IL-18 concentrations differed significantly between the subgroups (p = 0.0010, ϵ² = 0.096). Mean values were 80.50 ± 87.03 pg/mL (subgroup P), 48.01 ± 62.03 pg/mL (subgroup S), 40.45 ± 58.36 pg/mL (subgroup B), and 20.92 ± 31.73 pg/mL (group Z). *Post-hoc* analysis demonstrated significantly lower IL-18 levels in controls compared with groups P (p = 0.002) and S (p = 0.017).

IL-37 concentrations also differed significantly between subgroups (*p < 0.0001*, ϵ² = 0.111). Mean concentrations were 43.87 ± 53.47 pg/mL (subgroup P), 31.31 ± 35.22 pg/mL (subgroup S), 18.29 ± 28.90 pg/mL (subgroup B), and 7.17 ± 14.86 pg/mL (group Z). Controls differed significantly from groups P (p < 0.005) and S (p < 0.0001).

### 3.6 Correlation analysis

**Figure 4:**
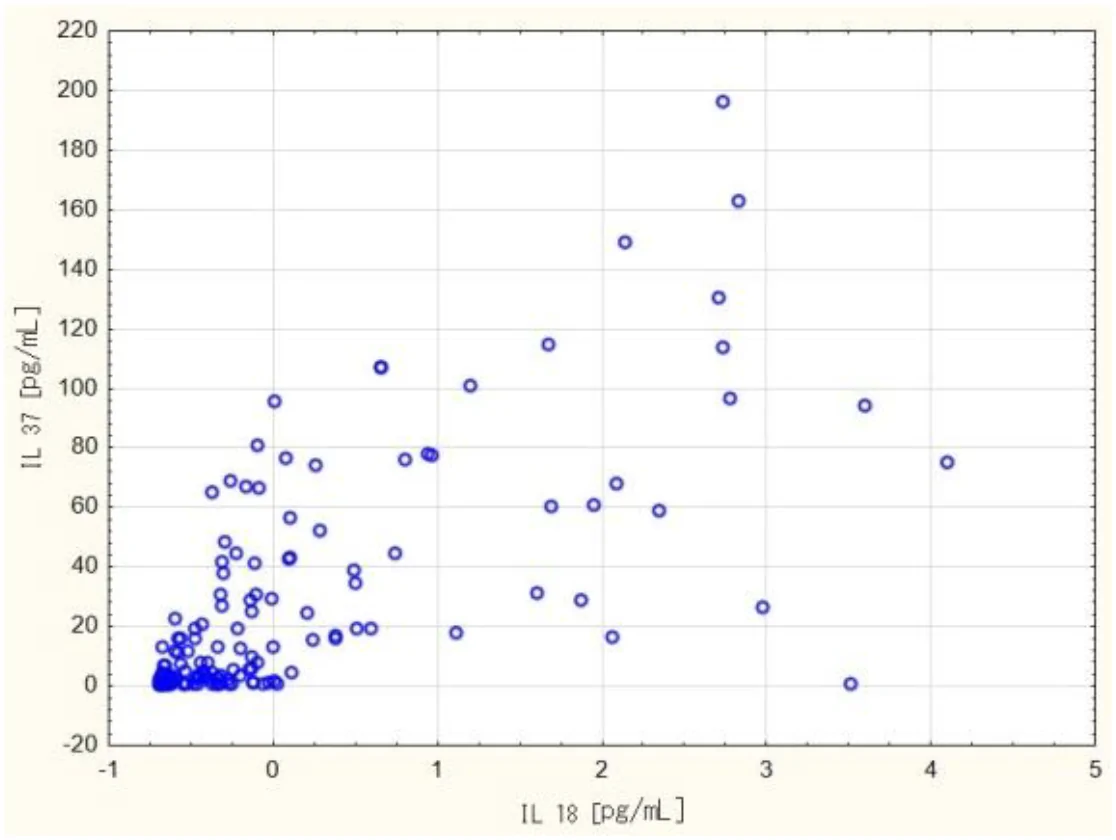
Correlation between IL-18 and IL-37 serum concentration. Spearman’s rank correlation coefficient: ρ = 0.751, *p* < 0.001.

A statistically significant positive association in the correlation analyses between IL-37 and IL-18 was observed using Spearman’s rank correlation coefficient (ρ = 0.751, t(162) = 14.46, p < 0.001), indicating a strong monotonic relationship between the variables. Consistent results were obtained using additional non-parametric measures. Goodman–Kruskal Gamma showed a significant positive association (γ = 0.578, Z = 10.76, p < 0.001), as did Kendall’s Tau (τ = 0.566, Z = 10.76, p < 0.001). Across all applied methods, the relationship between interleukin 37 and interleukin 18 remained statistically significant and consistently positive (**Figure 4**).

### 3.7 Multivariable analysis

In the multiple linear regression analysis, high seizure frequency (≥8 seizures/month) was independently associated with higher IL-18 concentrations (B = 27.66, 95% CI: 2.55–52.76; β = 0.297; *p* = 0.031). In addition, male sex was independently associated with lower IL-18 concentrations (B = −13.57, 95% CI: −26.59 to −0.55; β = −0.194; *p* = 0.041). No significant associations were observed for time since the last seizure, developmental delay, or seizure-free status (all *p* > 0.05) (**Table 2**).

**Table 2:** Multiple linear regression analysis of factors associated with IL-18 concentration.

Predictor	B (95% CI)	Standardized β	P-value
Time since the last seizure (days)	0.011 (-0.032 to 0.055)	0.081	0.604
Delayed developmental milestones	5.63 (-9.35 to 20.62)	0.076	0.458
High seizure frequency (≥8 seizures/month)	27.66 (2.55 to 52.76)	0.297	0.031
Seizure-free group	-5.76 (-26.73 to 15.21)	-0.069	0.587
Male sex	-13.57 (-26.59 to -0.55)	-0.194	0.041

B, unstandardized regression coefficient; β, standardized regression coefficient; CI, confidence interval.

In the multiple linear regression analysis, high seizure frequency (≥8 seizures/month) was independently associated with higher IL-37 concentrations (B = 9.76, 95% CI: 0.60–18.91; β = 0.217; p = 0.037). In contrast, intellectual disability was associated with lower IL-37 concentrations (B = −8.51, 95% CI: −16.82 to −0.21; β = −0.209; p = 0.045). Absence of carbamazepine treatment showed a trend toward lower IL-37 concentrations, although this association did not reach statistical significance (p = 0.071). Time since the last seizure was not significantly associated with IL-37 levels (**Table 3**).

**Table 3:** Multiple linear regression analysis of factors associated with IL-37 concentration.

Predictor	B (95% CI)	Standardized β	P-value
Time since the last seizure (days)	-0.014 (-0.030 to 0.002)	-0.171	0.091
High seizure frequency (≥8 seizures/month)	9.76 (0.60 to 18.91)	0.217	0.037
No carbamazepine treatment	-8.15 (-17.01 to 0.71)	-0.169	0.071
Intellectual disability	-8.51 (-16.82 to -0.21)	-0.209	0.045

B, unstandardized regression coefficient; β, standardized regression coefficient; CI, confidenceinterval.

### 3.8 Gene polymorphisms

The distribution of IL-18 −137G/C genotypes (GG 54.7%, CC 14.7%, GC 27.6%) and IL-37 genotypes (GG 25.3%, AG 45.3%, AA 26.5%) did not differ significantly between the subgroups. Furthermore, no significant associations were observed between either polymorphism and serum concentrations of IL-18 or IL-37.

## 4 Discussion

### 4.1 Main findings

The present study demonstrated that both serum IL-18 and IL-37 are significantly elevated in paediatric epilepsy. The data suggest an association between elevated peripheral cytokine levels and seizure burden. Previous studies have demonstrated elevated serum IL-18 levels in adult patients with epilepsy. However, evidence supporting these findings in paediatric population was scarce.

### 4.2 Role of IL-18 in epilepsy

Both interleukins belong to the IL-1 family but their biological roles appear divergent, with IL-18 exerting predominantly pro-inflammatory effects, while IL-37 acting as an anti-inflammatory regulator in immune responses (8).

IL-18 plays a broad role in immune regulation and has been studied in multiple inflammatory and autoimmune diseases (17). Elevated IL-18 levels have been linked to excitotoxic damage in the hippocampus (18). However, experimental studies also suggest that IL-18 may exert neuroprotective effects under certain conditions, for example by enhancing blood–brain barrier integrity (19, 20). Together, these findings indicate a complex and context-dependent role of IL-18 in epilepsy (13, 18–20).

Mochol et al. were the first to report increased serum levels of IL-18 and IL-18BP in patients with epilepsy, suggesting that IL-18 may act as a mediator of disease activity (13). In contrast to our paediatric cohort, their study included adults with stable and well-controlled epilepsy. Subsequent work by Dündar et al. confirmed elevated IL-18 levels in adult epilepsy patients compared with controls (21). Moreover, Liang et al. demonstrated higher serum concentrations of free IL-18 in patients with neuropsychiatric systemic lupus erythematosus presenting with seizures, supporting a potential role of bioactive IL-18 in seizure-related neuroinflammation (22). More recently, Nazli et al. demonstated markedly elevated IL-18 serum levels in adult patients with drug-resistant epilepsy increased progressively with seizure frequency (23). These findings further suggest that IL-18 may be associated with seizure burden and inflammatory processes underlying epilepsy, rather than simply with the diagnosis of epilepsy.

### 4.3 Role of IL-37 in inflammatory and autoimmune disorders

Ongoing research on chronic inflammatory and autoimmune disorders demonstrates that IL-37 expression is frequently increased in parallel with disease activity and elevated pro-inflammatory factors, including cytokines of the IL-1 family (24–26). Increased circulating or tissue levels of IL-37 have been consistently reported in conditions such as systemic lupus erythematosus, rheumatoid arthritis and inflammatory bowel disease and are often associated with markers of ongoing inflammation rather than clinical remission (15, 24, 27–30). These observations suggest that IL-37 levels may accompany increased inflammatory activity rather than indicate effective immune suppression.

### 4.4 Relationship between IL-18 and IL-37

In our analysis, a strong positive correlation was observed between IL-18 and IL-37 serum concentrations. Such a correlation may reflect a compensatory anti-inflammatory response, induced by increased inflammatory activity. It has been proposed that IL-37 upregulation may occur secondary to increased pro-inflammatory signalling mediated by IL-18, potentially representing an endogenous attempt to counterbalance excessive inflammation. Similar patterns have been reported in chronic inflammatory and autoimmune diseases, where anti-inflammatory cytokines are elevated along with pro-inflammatory factors, rather than replacing them (25–27). Although experimental studies suggest that IL-37 inhibits IL-18 signaling (25), its compensatory effect may be insufficient to fully neutralize IL-18-driven inflammation. Therefore, this interpretation should be considered a potential mechanism rather than evidence of a definitive biological relationship in paediatric epilepsy.

### 4.5 Effect of anti-seizure medications

In our data, VPA use was associated with lower serum IL-37 concentrations and CBZ use with higher IL-37 levels, while IL-18 concentrations remained unaffected by either drug. However, these findings should be interpreted with caution. Due to the fact that more than half of the studied patients received polytherapy, this may not necessarily reflect a direct drug effect, but rather the heterogeneity of the study group. Moreover, the choice of ASMs in clinical practice is related to epilepsy syndrome, seizure type, treatment response and previous therapeutic history. Therefore, the observed associations may reflect differences in disease characteristics rather than direct pharmacological effects of individual ASMs.

Nevertheless, beyond their established effects on neuronal excitability, ASMs are increasingly recognized as modulators of inflammatory pathways, including cytokine signaling within the central nervous system (31).

VPA demonstrated anti-inflammatory properties through inhibition of histone deacetylases (HDACs), leading to reduced expression of pro-inflammatory cytokines (31, 32). Experimental studies suggest that VPA may also downregulate IL-18 production indirectly by suppressing inflammasome activation, potentially contributing to the reduction of IL-18-mediated neuroinflammatory signalling (33, 34). Data regarding the direct influence of ASMs on IL-37 are currently limited. Lower IL-37 concentrations observed in VPA-treated patients may reflect reduced upstream inflammatory signalling and a diminished need for compensatory IL-37 induction, rather than a direct inhibitory effect of the drug on IL-37 expression. However, our findings represent a relatively large cohort of epilepsy patients and support the observations mentioned above. At the same time, this interpretation remains contradictory to recent reports suggesting increased or unchanged IL-37 expression under anti-inflammatory conditions (35–37) and should therefore be interpreted with caution.

To date, Mochol et al. are among a few researchers to have identified a significant association between IL-18 and specific ASMs. Their findings indicated that the IL-18/IL-18BP ratio was notably increased in patients treated with CBZ, remained unchanged in those receiving lamotrigine, and decreased following levetiracetam treatment (13). The observation by Mochol et al. of an increased IL-18/IL-18BP ratio in CBZ-treated patients suggests a shift towards enhanced IL-18 bioactivity. However, this does not necessarily imply a direct pro-inflammatory effect of CBZ.

### 4.6 Clinical interpretation and biomarker potential

The lack of correlation between IL-18 and IL-37 levels and time since the last seizure suggests that the cytokine alterations cannot be readily explained by the timing of the most recent seizure. This observation is consistent with previous studies reporting alterations in inflammatory cytokine levels in patients with epilepsy (38–41). However, the cross-sectional design of our study limits the ability to determine whether these inflammatory changes are sustained over time.

Elevated serum levels of IL-18 and IL-37 in epilepsy may represent a promising candidate biomarker associated with seizure burden, although further validation studies are required.

### 4.7 Genetic findings

Gene polymorphisms in cytokine genes may influence inflammatory responses through altered transcriptional activity. The IL-18 gene contains several promoter polymorphisms, including the 137 G/C variant, which have been associated with susceptibility to autoimmune diseases in different populations (42–44). Our data did not reveal any association between the IL-18–137 G/C polymorphism and seizure burden. Similarly, although IL-37 gene variants have been linked to autoimmune disease susceptibility (24), no relationship between IL-37 genotype and seizure burden was identified in our cohort. These findings suggest that the analyzed polymorphisms do not substantially contribute to interindividual variability in IL-18 or IL-37 levels in this paediatric cohort; however, larger studies are required to clarify the potential genetic determinants of immune dysregulation in this condition.

Immunological markers such as IL-18 and IL-37 are not intended to replace standard clinical assessment, although they may provide complementary information regarding overall disease burden and the presence of neuroimmune activation.

## 5 Limitations

Several limitations of this study should be acknowledged. The cross-sectional design limits the assessment of temporal changes in cytokine levels and prevents conclusions regarding causal relationships between inflammation and epilepsy progression. Serum cytokine levels may not fully reflect inflammatory processes in the central nervous system. However, peripheral measurements are commonly used and remain clinically relevant in biomarker studies. In addition, the heterogeneity of epilepsy etiologies and ASMs use may have affected cytokine levels. Furthermore, the seizure-frequency threshold used for study stratification has not been externally validated and the analyses did not include correction for multiple comparisons, ROC analysis or predictive modelling.

Further longitudinal studies in larger and more homogeneous patient groups are needed to confirm these results.

## 6 Future directions

Longitudinal assessment of IL-18 and IL-37 levels is needed to better understand their relationship with treatment response and epilepsy progression, including their potential value in predicting drug resistance. In addition, analysis of these cytokines in the cerebrospinal fluid and integration of inflammatory biomarkers with neuroimaging and genetic data may provide deeper insight into their involvement in epilepsy progression.

## 7 Conclusions

The study demonstrated that serum IL-18 and IL-37 concentrations are significantly elevated in paediatric patients with epilepsy, with the highest levels observed in patients with greater seizure burden. The strong positive correlation between these cytokines may reflect coordinated inflammatory regulation.

The IL-18/IL-37 axis may represent a promising candidate biomarker associated with seizure burden in paediatric epilepsy. However, these findings should be interpreted in the context of the study’s cross-sectional design and the heterogeneity of the patient cohort. Further independent longitudinal validation studies are required to clarify the temporal dynamics of these cytokines and to determine their clinical utility in assessing seizure burden and predicting treatment response.

## Data Availability

The data is located in the repository MOST Wiedzy, DOI 10.34808/s980-cj88.
